# Central Pathways Integrating Metabolism and Reproduction in Teleosts

**DOI:** 10.3389/fendo.2014.00036

**Published:** 2014-03-25

**Authors:** Md. Shahjahan, Takashi Kitahashi, Ishwar S. Parhar

**Affiliations:** ^1^Brain Research Institute, School of Medicine and Health Sciences, Monash University Malaysia, Petaling Jaya, Malaysia

**Keywords:** neuropeptide, metabolism, energy balance, fish, reproduction

## Abstract

Energy balance plays an important role in the control of reproduction. However, the cellular and molecular mechanisms connecting the two systems are not well understood especially in teleosts. The hypothalamus plays a crucial role in the regulation of both energy balance and reproduction, and contains a number of neuropeptides, including gonadotropin-releasing hormone (GnRH), orexin, neuropeptide-Y, ghrelin, pituitary adenylate cyclase-activating polypeptide, α-melanocyte stimulating hormone, melanin-concentrating hormone, cholecystokinin, 26RFamide, nesfatin, kisspeptin, and gonadotropin-inhibitory hormone. These neuropeptides are involved in the control of energy balance and reproduction either directly or indirectly. On the other hand, synthesis and release of these hypothalamic neuropeptides are regulated by metabolic signals from the gut and the adipose tissue. Furthermore, neurons producing these neuropeptides interact with each other, providing neuronal basis of the link between energy balance and reproduction. This review summarizes the advances made in our understanding of the physiological roles of the hypothalamic neuropeptides in energy balance and reproduction in teleosts, and discusses how they interact with GnRH, kisspeptin, and pituitary gonadotropins to control reproduction in teleosts.

## Introduction

A close connection between energy balance and reproduction has been well documented in mammals (1). Energy balance is maintained by a process that controls food consumption, energy expenditure, and energy storage. A number of hypothalamic neuropeptides including orexin, ghrelin, neuropeptide-Y (NPY), melanin-concentrating hormone (MCH), pituitary adenylate cyclase-activating polypeptide (PACAP), proopiomelanocortin (POMC)-derived peptides, cholecystokinin (CCK), chicken gonadotropin-releasing hormone-II (cGnRH-II), 26RFamide (26RFa), galanin (GAL), and cocaine- and amphetamine-regulated transcript (CART) have been implicated in the regulation of feeding behavior and energy balance. On the other hand, peripheral hormones such as leptin and ghrelin provide information about the availability of stored metabolic foods.

Initiation of reproduction is affected by the amount of body energy reserves and is responsive to diverse metabolic factors. The neuroendocrine mechanisms responsible for the association between energy balance and fertility are represented by metabolic hormones and neuropeptides that affect the hypothalamic center controlling the expression and release of gonadotropin-releasing hormone (GnRH) (2, 3). Therefore, adequate body energy stores are crucial for full activation of the hypothalamus–pituitary–gonadal (HPG) axis at puberty and its proper functioning in adulthood (4). Generally high amount of food supply favor reproduction, while low food supply inhibits the reproductive system (1). During energetic challenges, the physiological mechanisms that partition energy into various activities tend to favor the processes for the survival of the individual over the processes for growth, longevity, and reproduction (5). Therefore, the reproductive system is suppressed by energetic challenges. At the same time it is also true that when the reproductive system is highly activated, animal primates reproduction rather than feeding. Many factors such as starvation, eating disorders, excessive exercise, cold exposure, and lactation act on both food intake and reproduction by increasing hunger and/or food ingestion and by suppressing reproductive processes (5, 6).

Most feeding-related neuropeptides in mammals have also been identified in fish species (7), suggesting that the regulatory system of feeding has been well conserved from fish to mammals. On the other hand, as the links between energy balance and reproduction have been demonstrated in several vertebrates (8), this might also exist in teleosts. Indeed, seasonal changes in feeding often coincide with spawning migration and reproduction in fish, suggesting association between nutrition and reproduction (9).

This review focuses on the role of the neuropeptides that regulate feeding and energy balance on reproduction in teleosts, and discusses if the metabolic control of reproduction is conserved from fish to mammals.

## Regulation of Reproduction in Teleosts

In teleosts, as in other vertebrates, reproduction is coordinated by the HPG axis. The hypothalamus produces GnRH, which regulates the synthesis and release of gonadotropins (GTHs), follicle-stimulating hormone (FSH), and luteinizing hormone (LH), from the pituitary. The GTHs act on the gonads to stimulate gonadal development through the secretion of sex steroid hormones. These steroids, in turn, feedback to the brain and the pituitary to complete the HPG axis and to regulate the reproductive cycle (10, 11). Thus, hypothalamic GnRH is considered as the key player in the regulation of reproduction in teleosts. Furthermore, recent findings of kisspeptin and gonadotropin-inhibitory hormone (GnIH) added new players in the reproductive system, which stimulate and inhibit mostly GnRH neurons, respectively.

### Gonadotropin-releasing hormone (GnRH)

In the early 1970s, two research groups simultaneously reported the isolation of a LH-releasing factor from the hypothalamus of pigs and sheep (12, 13), and named it LH-releasing hormone (LHRH). Later, this decapeptide was also found to stimulate FSH release, and accordingly re-named GnRH. The GnRH isolated from mammals is also functional in fish, and stimulates the release of GTH in the carp (14). The first fish GnRH was identified in salmon, and named as salmon GnRH (sGnRH) (15). To date, 15 different forms of GnRH have been identified in vertebrates (13, 15–25), among them 10 original forms in fish species: salmon, sea bream, whitefish, medaka, catfish, herring, dogfish, and lamprey (lamprey I, II, and III). Most vertebrates possess two, and some teleosts have three, forms of GnRH in the brain (23, 25–31). Based on phylogenetic analysis, recent classification defines the species-specific (hypophysiotropic) form as GnRH1, while the most evolutionarily conserved chicken GnRH-II as GnRH2 (32). The third form is GnRH3 (33), which is present only in the brain of certain teleost species (31, 34).

Distribution of three different forms of GnRH in the brain was first reported in a perciform fish, the sea bream (35). GnRH1 neurons are generally present in the region from the ventral forebrain–preoptic area (POA) to basal hypothalamus, whereas GnRH2 neurons are restricted to the dorsal mesencephalon. GnRH3 neurons are located in the caudal-most olfactory bulb as a ganglion and along the terminal nerve in most fish species that possess three GnRH forms (31, 36). On the other hand, in the sea bream and the European sea bass, the distribution of GnRH1 and GnRH3 cells overlap in the olfactory bulbs, ventral telencephalon, and POA (37–40). Similar results were reported in several other fish species (41–45). In the sea bass brain, GnRH1 neuronal fibers are observed in the ventral surface of the forebrain, associated with the ventral telencephalon, POA, and the hypothalamus, whereas GnRH2 and GnRH3 neuronal fibers show profuse distributions throughout the brain (40).

The function of GnRH in the central regulation of LH release has been recognized in all orders of teleosts. Although the assay for FSH peptide is lacking for most fish species, studies in the rainbow trout (46–48) and the Coho salmon (49) show that GnRH also stimulates FSH release in salmonids. However, the different patterns of fiber projections of each GnRH form suggest different physiological function of each GnRH form in the brain (31). GnRH1 neurons are generally present in the ventral forebrain–POA–hypothalamus and send neuronal fibers directly into the pituitary, which represents its primary role in the stimulation of GTH secretion. The physiological significance of GnRH1 as a regulator of GTH secretion and gametogenesis has been established in several teleosts (28, 50–54).

GnRH2 neurons are exclusively present in the midbrain. The absence or low levels of GnRH2 peptide in the pituitary has been demonstrated in several *perciformes* (50, 51, 55–57) and pleuronectiformes species (28, 58), suggesting that GnRH2 is not directly involved in GTH secretion. Rather, its wide fiber projection throughout the brain suggests that GnRH2 has neuromodulatory functions (30). However, in some fish species including the goldfish, GnRH2 seems to act as a hypophysiotropic GnRH together with GnRH3 (59).

GnRH3 has been shown to control reproductive behaviors in several fish species. GnRH3 stimulates nest-building behavior in the male dwarf gourami (60), homing migration in the sockeye salmon (61), and aggressive and nest-building behaviors in the male Nile tilapia (62), which suggests probable neuromodulatory roles of GnRH3. The neuromodulatory role of GnRH3 was confirmed by electrophysiological studies in the retina of goldfish (63, 64) and olfactory receptor cells of the mudpuppy (65). The neuromodulatory function of GnRH3 has also been demonstrated in the rainbow trout (66, 67) and the dwarf gourami [reviewed by Oka (68)]. Fish species such as some salmonids and the zebrafish possess only two forms of GnRH (GnRH2 and GnRH3). In these species, GnRH3 expressed in the basal forebrain acts as a hypophysiotropic GnRH (45, 69–71).

### Kisspeptin

Kisspeptin is a neuropeptide that plays an important role in reproduction through the stimulation of GnRH neurons by activating GPR54 in mammals (72, 73). In teleosts, two kisspeptin genes, namely *kiss1* and *kiss2*, have been identified in several fish species (74–77), whereas placental mammals possess only the *kiss1* gene. Similarly, two kisspeptin receptor genes, named *kiss1r* and *kiss2r*, were also identified in several fish species (76, 78), suggesting two Kiss/Kissr systems in teleosts. However, this situation is not common among all fish species. Only one kisspeptin gene, *kiss2*, and one receptor, *kiss2r*, are present in some fish species including the Senegalese sole (79), orange-spotted grouper (80), grass puffer (81), and the Atlantic halibut (82), indicating that the *kiss1* and *kiss1r* genes have been lost during evolution in these species (82). Both *kiss1* and *kiss2* mRNAs are expressed in the brain and the gonads in several fish species (74, 76–78, 83). On the other hand, kisspeptin and kisspeptin receptor are also expressed in the fish pituitary, suggesting local actions of kisspeptin in the pituitary (76, 78, 81). In the medaka brain, two populations of *kiss1* neurons are found in the hypothalamus, one in the nucleus ventral tuberis (nVT) and the nucleus posterioris periventricularis (NPPv) (74, 84), while neurons in the dorsal zone of the periventricular hypothalamus (Hd) express *kiss2*. In the zebrafish all hypothalamic populations express *kiss2* mRNA (74). A recent study showed that zebrafish Kiss2 neuronal fibers are found widely in the subpallium, POA, ventral and caudal hypothalamus, and the mesencephalon (85). The fact that all three GnRH neuron types express kisspeptin receptors in the Nile tilapia (86) suggests that the role of Kiss2 neurons in the regulation of the HPG axis is via the activation of the GnRH systems. The *kiss1* neurons are exclusively localized in the habenula in the zebrafish (74), and send fibers only to the ventral part of the interpeduncular nucleus (85, 87). The habenula Kiss1 system is thus implicated in the modulation of serotonergic system rather than the HPG axis in the zebrafish (87).

The role of kisspeptin in the onset of puberty and sexual maturation is conserved among vertebrates including fish. In the zebrafish, both *kiss1* and *kiss2* mRNA levels are increased significantly at the start of the pubertal phase together with GnRH2 and GnRH3 mRNAs (74). Significant positive correlation is observed between the levels of *kiss2* mRNA and those of *gnrh1* mRNA during the spawning period in the grass puffer (81). Kiss2 but not Kiss1 stimulates GTH synthesis and release in the sea bass and the zebrafish (74, 75). Administration of Kiss2–10 peptide increases GnRH1 mRNA levels in the sexually mature female orange-spotted grouper (80), indicating that *Kiss2* most probably plays an important role in the regulation of reproductive functions through the stimulation of GnRH1 secretion.

The information of the interaction between kisspeptin neuronal fibers and GnRH cell bodies had been limited in teleosts due to the lack of specific antibody to kisspeptins. A recent study using an antibody to prepro-Kiss2 proved that Kiss2 neuronal fibers make close contacts with POA GnRH (GnRH3) neurons in the zebrafish (85), suggesting that Kiss2 directly act on GnRH neurons. Moreover, kisspeptin receptor expression in the three GnRH neuronal populations (86) in tilapia suggests that kisspeptin directly stimulates not only GnRH1 neurons to induce LH secretion, but also GnRH2 and GnRH3 neurons to activate other aspects of the reproduction such as sexual behavior.

### Gonadotropin-inhibitory hormone (GnIH)

GnIH or RFamide-related peptide (RFRP), which has a characteristic C-terminal LPXRFa motif (X = L or Q), is a hypothalamic neuropeptide that was originally identified from the quail as a neuropeptide that inhibits gonadotropin release from the pituitary (88). Extensive studies revealed that GnIH functions at the level of GnRH neurons and at the level of pituitary gonadotropes to suppress reproduction in avian and mammalian species [see reviews in Ref. (89, 90)]. GnIH in the teleost species has been named LPXRFamide peptide based on the amino acid sequence of the C-terminal motif. All precursors of teleost GnIH identified so far encode three GnIH orthologs (LPXRFa-1, -2, and -3), while only goldfish LPXRFa-3 has been purified as a mature peptide.

As in birds and in mammals, teleost GnIH neurons are located in the hypothalamus, in particular in the NPPv, and send neuronal fibers throughout the brain and to the pituitary (91, 92). The physiological function of teleost GnIH in the control of reproduction is complicated. *In vivo* studies using the goldfish show that GnIH decreases plasma LH levels as in avian and mammalian species (93, 94). On the other hand, GnIH significantly increases pituitary levels of mRNAs for LHβ and FSHβ in a reproductive state-dependent manner *in vivo*, whereas general suppression of LHβ and FSHβ mRNA levels is observed *in vitro* in a study (93). This differential *in vivo* effect of GnIH in different seasons can be explained by the differential action of GnIH on the gonads (95). GnIH does not affect plasma estradiol levels in the female goldfish, but increases plasma testosterone levels in the male goldfish (96). GnIH injections into the female goldfish suppress pituitary LHβ and FSHβ and hypothalamic GnRH mRNA levels (95). In addition, GnIH suppresses GnRH-induced increase in LHβ mRNA levels *in vitro* (95). Therefore, in the goldfish, the inhibition of the HPG axis at the level of hypothalamic GnRH neurons and pituitary gonadotropes appears as an evolutionarily conserved function of GnIH. On the contrary, goldfish GnIH peptides stimulate the synthesis and release of LH and FSH in cultured pituitary cells of the grass puffer and the sockeye salmon, respectively (92, 97). Therefore, as in mammals (98), the stimulatory or inhibitory action of GnIH in fish is probably species dependent or species-specific GnIH peptide might be necessary for an inhibitory action.

More recently, it has been shown that medaka LPXRFa-2 (GnIH-2) peptide decreases the firing frequency of non-hypophsiotropic terminal nerve GnRH3 neurons in the dwarf gourami (99). Since GnRH3 controls nest-building, aggression, and homing migration (60–62), GnIH-2 might negatively regulate reproductive behaviors.

## Metabolic Neuropeptides Involved in Reproduction

A number of hypothalamic neuropeptides have been identified in fish species (7), and found to be involved in the control of food intake as well as reproduction (Table 1). To understand the overall metabolic control of reproduction, the involvement of metabolic neuropeptides in the regulation of GnRH and GTHs must be taken into consideration. However, compared to mammals, the information related to the role of metabolic neuropeptides in the regulation of reproduction is still limited in fish.

**Table 1:** **Neuropeptides and their functions in representative fish species**.

Neuropeptide	Species	Function	Reference
Orexin	Goldfish	Increase food intake	(100)
	Ornate wrasse	Increase food intake	(101)
	Goldfish	Inhibit spawning behavior, decrease GnRH2 mRNA level	(102)
NPY	Goldfish	Increase food intake	(103)
	Rainbow trout	Increase food intake	(104)
	Puffer fish	Increase food intake	(105)
	Zebrafish	Increase food intake	(106)
	Goldfish	Stimulate GnRH and LH release	(107, 108)
	Common carp	Increase plasma LH level	(109)
	Sea bream	Stimulate GnRH release	(110)
	Sea bass	Increase plasma LH level	(111)
PACAP	Goldfish	Decrease food intake	(112)
	Goldfish	Stimulate LH release	(113, 114)
	Tilapia	Stimulate GTH subunit mRNA expression	(115)
	Blue gourami	Stimulate FSHβ mRNA expression	(116, 117)
GnRH2	Goldfish	Decrease food intake	(102, 118)
	Zebrafish	Decrease food intake	(119)
	Goldfish	Stimulate LH release	(120)
26RFa	Mouse	Increase food intake	(121)
	Goldfish	Increase plasma LH level	(122)
Galanin	Goldfish	Increase food intake	(123)
	Tench	Increase food intake	(124)
	(Rat)	Stimulate GnRH release	(125)
MCH	Goldfish	Decrease food intake	(126)
	Goldfish	Stimulate LH release	(127)
α-MSH	Goldfish	Decrease food intake	(128)
	Rainbow trout	Decrease food intake	(129)
	(Mouse)	Stimulate GnRH neurons	(130)
CART	Goldfish	Decrease food intake	(131, 132)
	(Rat)	Stimulate GnRH release	(133, 134)
CCK	Goldfish	Decrease food intake	(135)
	Goldfish	Stimulate LH release	(136)
Nesfatin-1	Goldfish	Decrease food intake	(137, 138)
	Goldfish	Decrease plasma LH level	(139)
Leptin	Goldfish	Decrease food intake	(140)
	Rainbow trout	Decrease food intake	(141)
	Sea bass	Stimulate LH release	(142)
	Rainbow trout	Stimulate LH release	(143)
Ghrelin	Goldfish	Increase food intake	(144–146)
	Rainbow trout	Decrease food intake	(147)
	Goldfish	Stimulate LH release	(148)
	Common carp	Stimulate LH release	(149)

### Orexin

Orexin has two well conserved molecular forms, a 33-amino acid peptide known as orexin A (OXA) and a 28-amino acid peptide known as orexin B (OXB) derived from the same precursor [see review in Ref. (150)]. Orexin was first identified as a ligand of an orphan receptor, and consequently found to stimulate feeding in mammals (151). The orexin’s orexigenic action is also observed in teleosts, including the goldfish and the ornate wrasse (101, 152).

In mammals, orexin is known to stimulate the HPG axis via GnRH secretion (153–155). In the goldfish, an interaction between orexin and hypophysiotropic GnRH (GnRH2) has also been proposed. Intracerebroventricular administrations of OXA inhibit spawning behavior and lower GnRH2 mRNA levels, while treatment with GnRH decreases OXA mRNA levels (102). These results suggest that, unlike in mammals, orexins might act as inhibitory agents in the control of GnRH at least in some fish species. In addition, OXA is detected in the pituitary of the medaka (156) and the Japanese sea perch (157), whilst OXB is detected in the pituitary of the Nile tilapia (158), suggesting orexin’s local action at the level of pituitary. Thus orexin, an orexigenic neuropeptide, inhibits the HPG axis at the hypothalamus GnRH level and possibly also at the pituitary level, in fish.

### Neuropeptide-Y (NPY)

NPY which is composed of 36 amino acid residues, was first identified in the porcine brain (159), and was found to function as a powerful appetite enhancer in mammals (160). In fish species, NPY also show powerful orexigenic activity in the goldfish (103, 123, 161–164), trout (104), puffer fish (105, 165), and the zebrafish (106).

Centrally or peripherally injected NPY increases plasma LH levels in the goldfish, common carp, rainbow trout, and in the sea bass (107, 109, 111), indicating that NPY stimulates teleost reproduction as was shown in mammals (166).

In the brown trout (167) and the rainbow trout (168), NPY neuronal fibers project to the areas where hypophysiotropic GnRH neurons exist, particularly in the ventral telencephalon, POA, and in the basal hypothalamus. Furthermore, double immunolabeling reveals close appositions of NPY fibers with GnRH cells in the POA of the ayu (*Plecoglossus altivelis*) (169) and the Siberian sturgeon (170), suggesting the direct action of NPY in the regulation of GnRH neurons. Indeed, NPY stimulates GnRH release *in vitro* in the goldfish (108) and in the sea bream (110).

Neuropeptide-Y also regulates the HPG axis at the level of pituitary. *In vitro* treatment with NPY stimulates LH release from pituitary cells in the goldfish (107) and increases LHβ and GTHα, but not FSHβ mRNA levels in the tilapia pituitary (115). In addition, NPY fibers make close appositions on LH cells in the catfish pituitary (171).

These findings provide strong support for the stimulatory role of NPY in fish reproduction at the levels of hypothalamic GnRH and pituitary LH cells.

### 26RFamide (QRFP)

26RFamide is a 26-amino acid peptide, and was first isolated from the frog brain (121). In teleosts, 26RFa has been identified only in the goldfish (172). The 26RFa gene is highly expressed in the hypothalamus, and relatively less in the optic tectum-thalamus and in the testis (122). 26RFa and its mammalian homolog QRFP act as an orexigenic hormone in birds, mice (121, 172–174), and probably in fish (122).

The role of 26RFa has been implicated in the integration of metabolism and reproduction in vertebrates, including fish [see review in Ref. (89)]. In mammals, 26RFa stimulates LH and FSH release in rats *in vivo* and *in vitro* (pituitary culture) (175). In teleost, intraperitoneal injections of 26RFa significantly increase plasma LH levels in the goldfish (122). On the other hand, *in vitro* treatment with 26RFa shows no effects on LH release from pituitary cells. These facts indicate that 26RFa might act on the stimulation of the HPG axis through GnRH1 release in fish.

### Galanin (GAL)

GAL is a 29-amino acid peptide, expressed in the central nervous system and in the intestine. GAL stimulates feeding in the goldfish (123) and the doctor fish tench (124), indicating that GAL acts as an orexigenic hormone in fish as in mammals (176, 177).

Involvement of GAL in the control of HPG axis is evidenced in mammals. In rodents and humans, GAL neuronal fibers make close appositions with GnRH1 neurons (178–180), and GnRH neurons express a GAL receptor Gal-R1 in the rat (181). In fact, GAL stimulates *in vitro* GnRH release in rats (125). These data indicate that GAL is involved in the control of reproduction at the level of GnRH neurons.

In fish, there are no studies that demonstrated the role of GAL in the control of reproduction. However, close appositions of GAL fibers with gonadotropes in the proximal pars distalis (PPD) are seen in the sea bass (182). Similarly, fiber projections of GAL neurons in the PPD are observed in the rainbow trout (183), sea bream (184), and Senegalese sole (185), while no GAL fibers are observed in the pituitary of the Siberian sturgeon (170). Therefore, GAL might modulate the HPG axis at the pituitary level at least in some fish species.

### GnRH2

As mentioned before, among the different forms of GnRH, neuronal fibers of GnRH2 (also known as chicken GnRH-II) are widely distributed in the vertebrate brain. In an insectivore, the musk shrew, GnRH2 stimulates sexual behavior and seduces food intake (186, 187), indicating that GnRH2 plays a role in connecting reproductive function and feeding regulation.

In fish species, the suppressive effect of GnRH2 on feeding has also been confirmed. Food consumption is significantly decreased by intracerebroventricular injections of GnRH2 but not GnRH3 in a dose dependent manner in the goldfish (102, 118) and the zebrafish (119).

GnRH2 also has effects on sexual behavior of fish. In the goldfish, GnRH2 stimulates reproductive behavior (188). Furthermore, there is a strong positive correlation between spawning behavior and GnRH2 gene expression (189), suggesting stimulatory role of GnRH2 in reproductive behavior. GnRH2 is also detected in the goldfish pituitary (190) and induces LH release *in vitro* (120). Positive correlation between the pituitary GnRH2 levels and gonadal development is also observed in the striped bass (51), suggesting that it also have a hypophysiotropic role in some fish species. In the grass puffer, the amount of GnRH2 mRNA is slightly higher in the post-spawning females compared to spawning female (191). Therefore, GnRH2 may have different physiological roles depending on the physiological conditions of the fish.

### Pituitary adenylate cyclase-activating polypeptide (PACAP)

PACAP was first isolated from the rat hypothalamus (192). PACAP is an anorexigenic factor in various vertebrates, including rodents (193), chicks (194, 195), and fish (196). To date, the role of PACAP in feeding has been studied only in one fish species, the goldfish.

PACAP increases plasma LH levels *in vivo* in the goldfish (197). *In vitro* studies showed that the stimulatory effect of PACAP on LH release is exerted at the level of the pituitary (113, 114). PACAP also stimulates the levels of GTH subunit mRNAs and FSHβ mRNA in the pituitary of tilapia (115) and in the female blue gourami (116, 117), respectively. Dense projection of PACAP nerve terminals is seen in the pars distalis of the pituitary, where gonadotropes are localized, in the goldfish (198) and in the European eel (199). The expression of PACAP receptor in the pituitary is also observed in the goldfish (197). Therefore, PACAP stimulates GTH secretion in fish pituitary.

### Melanin-concentrating hormone (MCH)

MCH is a cyclic peptide, originally isolated from the pituitary of the chum salmon as a hormone involved in body color change (200). In the winter and barfin flounders, fasting stimulates hypothalamic expression of MCH (201, 202), suggesting that MCH acts as an orexigenic hormone as in mammals (203, 204). However, MCH acts as an anorexigenic hormone in the goldfish (105, 126, 205, 206). Therefore, like ghrelin, MCH acts as an orexigenic and anorexigenic neuropeptide depending on the fish species, although its orexigenic action in fish has to be confirmed.

In mammals, MCH modulates LH secretion in an estradiol-dependent manner [see a review in Ref. (207)]. The close appositions between MCH fibers and hypothalamic GnRH neurons (208, 209) and the expression of MCH receptors in GnRH neurons (209) suggest the direct action of MCH on GnRH neurons in mammals. MCH also acts at the pituitary level to modulate the release of LH (210). In teleosts, an *in vitro* study showed that salmon MCH stimulates the release of LH in a dose response manner from dispersed pituitary cells in the goldfish, suggesting a direct action of MCH on LH cells (127). Whether MCH acts on GnRH neurons in fish as in mammals remains unknown.

### α-Melanocyte stimulating hormone (α-MSH)

α-MSH is one of melanocortins and derived from a precursor peptide encoded by the POMC gene (211). Among melanocortins and their receptors, α-MSH and melanocortin receptor 4 (MC4R) are involved in the control of food intake in vertebrates including fish. α-MSH or MC4R agonist inhibits food intake in the goldfish (128, 212) and in the rainbow trout (129), suggesting that the α-MSH/MC4R system play a role in the anorexigenic regulation of feeding in fish as in mammals.

Although the α-MSH/MC4R system is known to play a stimulatory role in reproduction at the level of GnRH neurons in mammals [see a review in Ref. (130)], available information is limited in teleost. Projection of α-MSH fibers in the PPD of the pituitary and differential expression of POMC gene between sexually inactive and active fish in the zebrafish suggests that some of POMC-derived products are involved in the stimulation of fish reproduction (213).

### Cocaine- and amphetamine-regulated transcript (CART)

CART is an anorexigenic neuropeptide originally isolated from the rat brain (160, 214). In fish, CART might also act as anorexigenic hormone in the goldfish (131), winter flounder (215), cod (216), channel catfish (217), zebrafish (218), and in the Atlantic salmon (219).

In mammals, CART is involved in the control of GnRH neurons. CART stimulates GnRH pulsatile release in rats (133, 134). The existence of close appositions between CART fibers and hypothalamic GnRH neurons in the Siberian hamster suggests the effect of CART on GnRH neuronal activity is a direct action (220).

In the catfish, the projections of CART fibers are observed in the PPD of the pituitary (221). CART is also expressed in LH cells of the catfish pituitary but only during sexual maturation period (222), suggesting its local function in the sexual maturation process. However, it should be noted that while similar expression of CART in LH cells is observed in the rat pituitary, CART inhibits the release of prolactin but not GTHs (223). Thus, the role of CART in the fish pituitary has to be examined.

### Cholecystokinin (CCK)

CCK is found in the brain and in the gastrointestinal tract of various vertebrates. It has multiple biologically active forms, among which CCK-8 is the most abundant form in the brain (224). As in mammals, CCK has many physiological roles in fish, but functions primarily in the control of food intake as a satiety indicator (135).

In mammals, CCK decreases the pulse interval of GnRH release in goats (225). Furthermore, CCK implants into the POA, where GnRH neurons are located, increase the plasma levels of LH in rats (226). These data suggest that CCK acts at the levels of GnRH and stimulates reproduction. In fish, on the other hand, CCK seems to acts on the pituitary. An immunohistochemical study showed that CCK neurons innervate into the PPD of the pituitary and that CCK stimulates LH release *in vitro* in the goldfish (136).

### Nesfatin-1

Nesfatin-1, a nucleobindin-2 (NUCB2) encoded unmodified peptide, was first characterized in rats (227), and was shown to have anorexigenic actions in the goldfish (137, 138).

The number of studies about the function of nesfatin-1 in the control of reproduction is still limited. However, recent studies showed that nesfatin-1 acts as an inhibitory signal in the control of fish reproduction. Although nesfatin-1 plays a stimulatory role in LH secretion in rats (228), an intraperitoneal injection of nesfatin-1 decreases plasma levels of LH in the goldfish (139). At the same time, nesfatin-1 down regulates expression of GnRH, LHβ, and FSHβ genes, suggesting that the inhibitory action of nesfatin-1 takes place at the levels of GnRH neurons. Whether nesfatin-1 also functions at the level of the pituitary remains unclear.

## Peripheral Hormones Involved in Feeding, Metabolism, and Reproduction

### Leptin

Leptin is primarily produced by adipocytes of the white adipose tissue (229), and secreted into the blood circulation in proportion to the mass of body fat. The change in plasma leptin levels is detected by the hypothalamus and thereby it acts as a peripheral factor that signals nutritional status to the CNS [see review by Crown et al. (230)]. In teleosts including the goldfish and the rainbow trout, leptin functions as a peripheral signal to inhibit food intake (140, 141, 231, 232) as in mammals (233).

In mammals, leptin stimulates the HPG axis by promoting the synthesis and release of GnRH from the hypothalamus, and LH and FSH from the pituitary (234–236). In teleosts, leptin also stimulates the reproductive axis. Leptin increases *in vitro* LH release from the pituitary culture in the sea bass (142) and the rainbow trout (143). However, it should be noted that the stimulating effect of leptin on LH release is observed only on the pituitary samples from the fish in maturational stages. Furthermore, leptin expression levels increase with the onset of sexual maturation in the Arctic char (237) and the Atlantic salmon (238). Therefore, the role of leptin in sexual maturation seems to be conserved among vertebrate species.

### Ghrelin

As in mammals, ghrelin is highly expressed in the stomach and moderately in the brain (144, 239, 240), and is involved in appetite stimulation, energy balance, feeding, and metabolism [see reviews in Ref. (241, 242)]. Interestingly, the role of ghrelin in fish differs in different fish species. It acts as an orexigenic hormone in the goldfish (144–146) and probably in the sea bass (243) and the zebrafish (244). On the other hand, ghrelin acts as an anorexigenic hormone in the rainbow trout (147) and probably in the burbot (245, 246). The opposite effects of ghrelin on food intake can be explained by species-specific neural pathways mediating the effect of ghrelin (247). The variations in the role of ghrelin in feeding may reflect different regulatory mechanisms of feeding in different teleost species.

In fish species, ghrelin acts as a stimulatory factor in the reproduction, although ghrelin inhibits the HPG axis in mammals [reviewed by Tena-Sempere (248)]. Intracerebroventricular injection of ghrelin increases plasma LH levels in the goldfish (148), indicating its stimulatory action on the HPG axis. The increase of plasma LH levels is, however, small and slow compared to the increase of plasma GH levels. This suggests that the stimulatory effect of ghrelin on plasma LH levels is not through the action of ghrelin on hypothalamic GnRH. Actually, the highest levels of ghrelin receptor mRNA are observed in the sea bream and goldfish pituitary (249, 250). *In vitro* treatment with ghrelin stimulates LH release in the goldfish (148, 250) and in the common carp (149), while pituitary levels of mRNA for LHβ subunit is also increased. As no reports show fiber projections of hypothalamic ghrelin neurons into the pituitary in fish, ghrelin released from stomach/intestine might play a role in the LH secretion from the pituitary. Therefore, ghrelin might act as a stimulatory peripheral factor in reproduction at the level of pituitary, whereas its action on GnRH neurons is uncertain.

## Interactions between Metabolic Neuropeptides and the Reproductive System in the Control of Reproduction

**Figure 1:**
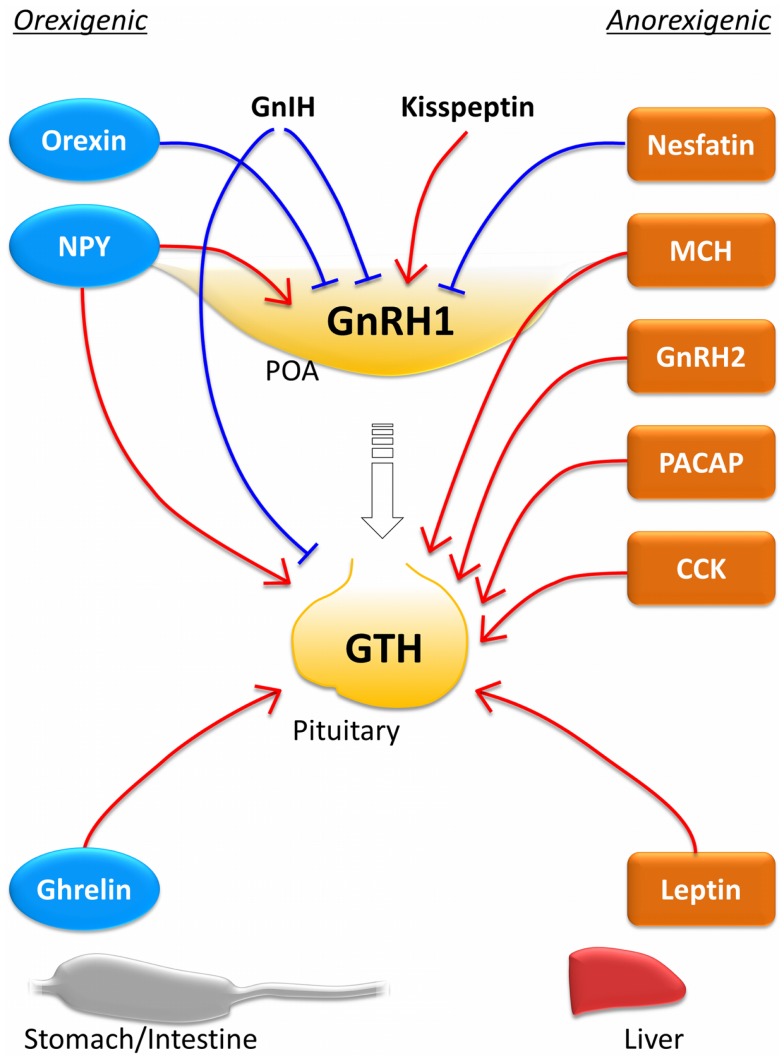
**Effects of central and peripheral metabolic hormones on the reproductive system**. The hypothalamus–pituitary axis receives many stimulatory and inhibitory inputs from central metabolic neuropeptide neurons and peripheral metabolic signals to control the reproductive system according to the energy status. The hormones indicated with blue circles are orexigenic hormones, while those with orange squares are anorexigenic ones. Red lines indicate stimulatory action and blue lines indicate inhibitory action. Most anorexigenic hormones stimulate gonadotropin secretion at the pituitary level. In the goldfish, NPY and ghrelin, which act as orexigenic hormones, also stimulate the reproductive system at the brain and pituitary levels. It should be noted that this figure is drew primarily based on the information obtained from the goldfish. Function of each metabolic hormone might differ in different species [e.g., Ghrelin functions as an anorexigenic factor in the rainbow trout (147) and GnIH stimulates GTH subunit mRNA expression in the puffer fish (97) and the sockeye salmon (92).]. CCK, cholecystokinin; GnIH, gonadotropin-inhibitory hormone; GnRH, gonadotropin-releasing hormone; MCH, melanin-concentrating hormone; NPY, neuropeptide-Y; PACAP, pituitary adenylate cyclase-activating polypeptide.

As shown in the above section, many metabolic neuropeptides are involved in the control of reproduction at the level of hypothalamic GnRH neurons and at the level of pituitary gonadotropes (Figure 1). Among these metabolic neuropeptides, NPY and nesfatin-1 function as inhibitory factors on GnRH neurons, while orexin stimulates GnRH neurons. It is interesting that NPY and orexin, which possess orexigenic activity, act on the reproductive system in an opposite manner in the goldfish. It suggests that different metabolic neuropeptides might play a role in the control of reproduction under different physiological conditions. It should be noted, however, that the inhibitory role of orexin on spawning behavior and GnRH gene expression might be the result of orexin action on non-hypophysiotropic GnRH system. In the goldfish brain, hypophysiotropic GnRH type is expressed not only in the hypothalamic population but also in the olfactory bulb and midbrain populations (190). Therefore, the inhibitory effect of orexin on the HPG axis need to be confirmed although it is clear that orexin has suppressive role in some aspects of reproduction.

At the pituitary level, it is evident that many metabolic neuropeptides including NPY, MCH, GnRH2, PACAP, and CCK stimulate LH secretion. In addition, peripheral metabolic signals such as ghrelin and leptin also stimulate LH secretion at the pituitary. The fact that most central neuropeptides and peripheral metabolic signals regulate the reproductive system indicates fundamental interaction between energy balance and reproduction, which is evolutionarily conserved from fish to mammal. However, both orexigenic and anorexigenic metabolic signals act as stimulatory factors in the reproductive system in fish. In mammals, feeding and reproduction are two alternatives in general. Therefore, orexigenic factors inhibit reproduction and anorexigenic factors stimulate reproduction [see review in Ref. (251)]. In fish species, on the other hand, a central orexigenic neuropeptide NPY and a peripheral orexigenic peptide ghrelin inhibit LH secretion in the goldfish and other species (Table 1). This indicates that metabolic regulation of the reproductive system in teleost is different from that in mammals, at least in some species.

Fish species have a variety of feeding and reproductive behaviors. For example, most salmonids and the winter flounder undergo a period of fasting just before the spawning season as a part of their normal physiology (252), whereas the goldfish do not have such fasting period. The halt of food intake during final maturation might require the differential usage of metabolic signals in these species.

Recently, kisspeptin has been proposed as a mediator of metabolic signals in the mammalian reproductive system, in particular on GnRH neurons [see reviews in Ref. (89, 253)]. In mice (254) and in the sheep (255), kisspeptin neurons in the arcuate nucleus possess leptin receptors, suggesting direct action of leptin on kisspeptin neurons. Furthermore, kisspeptin neurons receive innervations from other neurons that express leptin receptor (255). These facts suggest that leptin controls GnRH neurons through kisspeptin neurons *via* direct and indirect actions. Furthermore, kisspeptin neurons receive fiber projections from NPY and POMC neurons in mammals (255, 256). Therefore kisspeptin neurons might play an important role in the integration of metabolic signals to control the reproductive system. In teleost, fasting induces a significant increase in *kiss2* mRNA levels in the hypothalamus, as well as an increase in LHβ and FSHβ mRNA levels in the pituitary in the Senegalese sole (*Solea senegalensis*) (257), suggesting negative correlation between energy balance and reproduction. However, to our knowledge, there is no information available regarding direct evidence of metabolic regulation of kisspeptin neurons in fish.

In addition to its primary role in reproduction, GnIH stimulates food intake in chickens (258) and in rats (259, 260), suggesting its potential role to switch from reproduction to feeding. Close appositions of GnIH fibers with NPY, orexin, MCH, and POMC neurons in the sheep (261) indicate the involvement of several feeding regulatory pathways. However, there are no studies reporting metabolic regulation of GnIH neurons in vertebrates. On the other hand, GnIH is known to be regulated by stress, photoperiod, and gonadal steroids to suppress the reproductive system (89). Therefore, GnIH neurons might have a role in the modulation of feeding according to the environmental factors in mammals. Whether GnIH plays a similar role in teleosts requires more studies.

## Interactions among Neuropeptides to Control Feeding

To monitor the amount of energy stock, central metabolic neuropeptide neurons receive peripheral signals including leptin and ghrelin. For example, in mammals leptin receptor is expressed in many metabolic neuropeptide neurons including orexin, NPY, GAL, MCH, POMC, CART, and CCK neurons [see reviews in Ref. (262, 263)]. In fish species, leptin also affects several central neuropeptide neurons. Administration of leptin reduces NPY mRNA levels in the goldfish (140), grass carp (264), and in the rainbow trout (141, 265). On the other hand, leptin increases the mRNA levels of CCK and POMC, which are anorexigenic neuropeptides, in the goldfish (140) and the rainbow trout (141, 265), respectively. A recent study showed that leptin receptor knockout medaka exhibit higher levels of NPY mRNA before and after feeding and lower levels of POMC mRNA levels after feeding together with increased food intake (266). Therefore leptin’s anorexigenic effect might be mediated by these neuropeptides.

**Figure 2:**
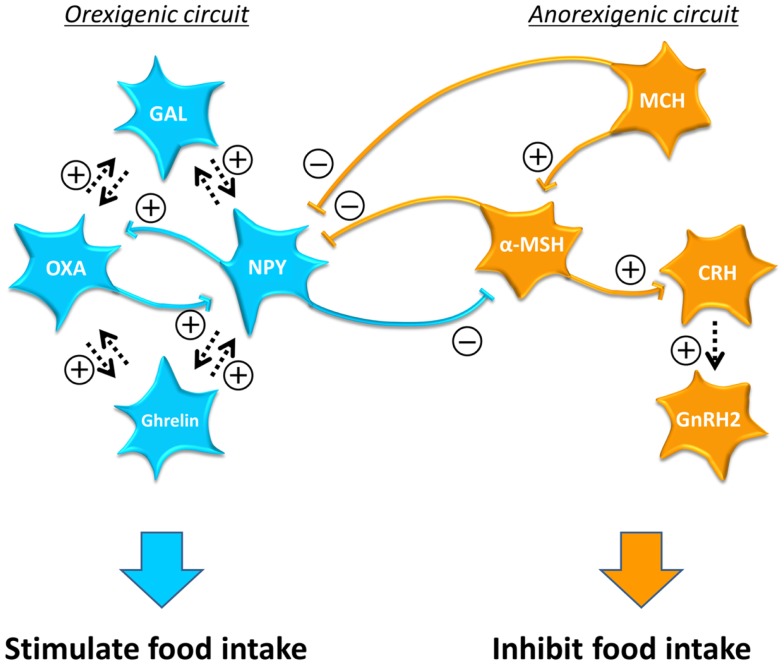
**Interaction between the orexigenic circuit and anorexigenic circuit and among neurons in each circuit in the goldfish**. Hormones in the orexigenic circuit are shown in blue and those in the anorexigenic circuit are shown in orange. The orexigenic and anorexigenic circuits are connected each other via inhibitory neuronal fiber projections to form an on-off switch. Reciprocal interaction is observed especially between neurons in the orexigenic circuit. Arrows with dotted lines indicate the interactions that have not been confirmed whether direct or indirect. The complex interaction among metabolic neuropeptides suggests that a change in one metabolic signal can affect the reproductive system through the action via other metabolic neuropeptides. Note that in the goldfish ghrelin secreted in the brain also function as an orexigenic signal. α-MSH, alpha-melanocyte stimulating hormone; CRH, corticotropin-releasing hormone; GAL, galanin; GnRH, gonadotropin-releasing hormone; MCH, melanin-concentrating hormone; NPY, neuropeptide-Y; OXA, orexin A; PACAP, pituitary adenylate cyclase-activating polypeptide.

Double immunostaining revealed interactions among orexigenic/anorexigenic neuropeptide neurons in teleosts, in particular in the goldfish (Figure 2). Among anorexigenic neuropeptide neurons, MCH neuronal fibers project to α-MSH neurons (206) and α-MSH neuronal fibers project to CRH neurons (267). Furthermore, a study using antagonists against α-MSH receptor and CRH receptor showed that anorexigenic action of MCH is mediated by α-MSH and CRH (268). In addition, GnRH2 mediates anorexigenic effect of α-MSH and CRH (269). These results suggest that the MCH–α-MSH–CRH–GnRH2 pathway suppresses food intake in the goldfish, although it is not known whether CRH directly acts on GnRH2 neurons.

Among orexigenic neuropeptide neurons, NPY and orexin neurons make reciprocal connections in fish as in mammals. NPY neuronal fibers make close appositions with orexin neurons, whereas orexin neuronal fibers make close appositions with NPY neurons in the NPPv in the goldfish (270). Furthermore, co-injections of OXA and NPY result in food intake higher than that observed in fish treated with NPY alone (132). These results indicate that orexins and NPY induce orexigenic actions by mutual signaling pathways in the CNS in teleost. Probably the reciprocal interaction between NPY and orexin functions as a positive-feedback system to maintain food intake.

Moreover, the orexigenic and the anorexigenic circuits are also connected with each other. α-MSH neuronal fibers make close appositions with NPY neurons, whereas NPY neuronal fibers project to α-MSH neurons in the goldfish (271). In addition, MCH neuronal fibers make close appositions with NPY neurons (272). These inhibitory inputs between the orexigenic and anorexigenic neurons might function as an on/off switch to decide whether eat or not eat by activating only one of the two circuits.

Studies using antagonists against of the receptors for metabolic neuropeptides further provided possible interaction among central metabolic neuropeptides in the goldfish. For example, GAL mediates the orexigenic action of orexin, and orexin mediates the orexigenic action of GAL (123). GAL also mediates NPY’s action on food intake and *vice versa* (123). Besides, orexin mediates central action of ghrelin in food intake and central ghrelin mediates the action of orexin (273). Furthermore, NPY mediates the orexigenic action of ghrelin (274). These results indicate complex neuronal interactions especially among central orexigenic neuropeptides. This complex neuronal network suggests that many central neuropeptide neurons function in a coordinated manner to regulate food intake. To fully understand the whole circuit that controls food intake, further information on the neuronal interaction among central metabolic neuropeptides have to be obtained.

In addition to the evident neuronal interactions in the goldfish, more combinations of neuronal interactions were reported in other fish species. In the masu salmon (275) and the Siberian sturgeon (170), NPY and GAL neurons make reciprocal connections. In the medaka, orexin and MCH neurons send neuronal fibers to each other (156). In the barfin flounder, reciprocal connection between orexin and MCH neurons and between α-MSH and MCH neurons was reported (156). In the rainbow trout, CRH mediates the anorexigenic action of ghrelin (147). These facts suggest that the interaction among central metabolic neuropeptides is really complicated. Therefore, more fiber projection studies together with the localization of the neuropeptide receptors are necessary to understand proper relationships among these neuropeptides that consist of the regulatory circuits of food intake.

## Perspectives

### Direct interactions among central metabolic neuropeptides and reproductive system

Significant amount of information about the relationship among orexigenic and anorexigenic neuropeptides have been accumulated, particularly in the goldfish. However, the knowledge of direct interactions among these neurons is still not enough to draw a complete diagram of the neuronal circuit to control food intake and reproduction in fish. In particular, metabolic regulation of kisspeptin and GnRH neurons are still unknown, while it is suggested from mammalian studies. Further fiber projection studies using double immunostaining and localization of the neuropeptide receptors in certain neuronal cell bodies need to be performed.

### Species differences

There are many differences in the regulatory mechanism of food intake and reproduction not only between mammals and fish but also between fish species. The significant difference between fish species might be the result of the adaptation to a wide range of feeding habits and reproductive strategies. Therefore we have to be careful to combine data obtained from different species.

### Sex and maturational stages

Several studies reported that the responses of the reproductive system to metabolic signals differ depending on the sex and the stage of sexual maturation. In fact, gonadal steroids modulate the effect of NPY on GnRH and LH release in the goldfish (276). Each study should use a particular sex and maturational stage to make comparison easy.

### Nutritional conditions

Animals might change the metabolic control of reproduction according to the available energy stock. For example, short term food limitation attenuates sexual motivation, while remaining energy stock still maintains activity of the HPG axis. On the other hand, long term food limitation depletes the energy stock and stops the HPG axis to prioritize the energy supply to the survival. Thus, feeding conditions and the timing of experiment might be important to obtain comparable data.

### Endocannabinoid system

The endocannabinoid system is involved in a variety of physiology including pain-sensation, mood, and memory. Importantly, both energy balance and reproduction are modulated by the endocannabinoid system. Endocannabinoids modulate several hypothalamic metabolic neuropeptides in mammals [see reviews in Ref. (277, 278)]. The endocannabinoid system also regulates food intake in fish (279, 280). In mammalian and non-mammalian vertebrates, the endocannabinoid system regulates hypothalamic GnRH neurons and pituitary LH cells directly and indirectly [see reviews in Ref. (281, 282)]. Interrelation among these systems might be an additional mechanism underlying the interaction between mood, stress, appetite, and reproduction.

## Conclusion

In summary, the cellular and molecular basis for the integration of feeding and reproduction involves a complex interaction of the reproductive system with metabolic neuropeptides and peripheral fuels. The metabolic neuropeptides, particularly orexin, NPY, PACAP, MCH, nesfatin, GnRH2, and CCK play an important role in the reproduction by either regulating GnRH neurons in the hypothalamus or by stimulating gonadotropes in the pituitary. Peripheral metabolic signals such as ghrelin and leptin also act on the pituitary to stimulate LH secretion. It should be, however, noted that compared to mammals, fishes show a great variety of feeding and reproductive habits. The variations of metabolic control of reproduction in different teleost species may reflect different requirement of energy status for reproduction in different species. Compared to mammals, fish represent a vast phylogenetic group, which shows a significant level of diversity with regards to morphology, ecology, behavior, and genomes (283). Thus, species differences in the neuroendocrine control of reproduction have to be taken into consideration in teleosts. In addition, more detailed studies about the interconnections among metabolic neuropeptide neurons, effects of sexual maturation, and nutritional conditions will provide more precise figure of the metabolic control of reproduction. Furthermore, differential control of multiple GnRH neuronal population by the neuropeptides and metabolic signals should be examined to elucidate their roles in different aspects of metabolic control of reproduction.

## Conflict of Interest Statement

The authors declare that the research was conducted in the absence of any commercial or financial relationships that could be construed as a potential conflict of interest.
